# Icephobic Gradient Polymer Coatings Deposited via iCVD: A Novel Approach for Icing Control and Mitigation

**DOI:** 10.1021/acsami.3c18630

**Published:** 2024-02-24

**Authors:** Gabriel Hernández Rodríguez, Mario Fratschko, Luca Stendardo, Carlo Antonini, Roland Resel, Anna Maria Coclite

**Affiliations:** † Institute of Solid State Physics, NAWI Graz, Graz University of Technology, 8010 Graz, Austria; ‡ Department of Materials Science, University of Milano-Bicocca, via R. Cozzi 55, 20125 Milano, Italy

**Keywords:** iCVD, icephobic, gradient polymer, coatings, anti-icing, icephobic surface design

## Abstract

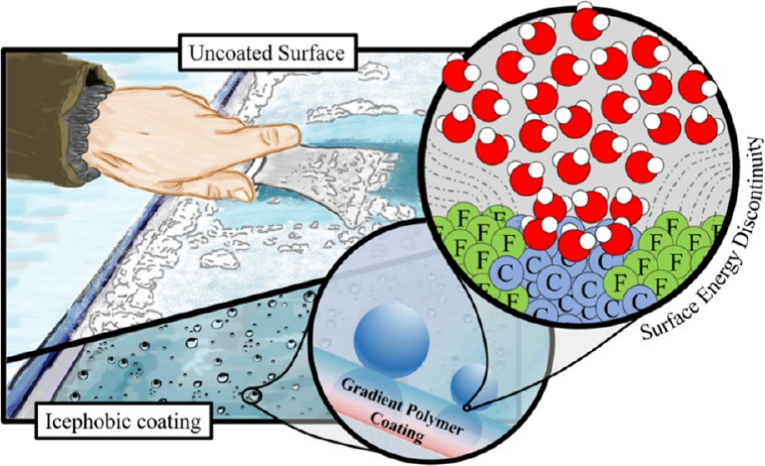

Materials against ice formation and accretion are highly desirable for different industrial applications and daily activities affected by icing. Although several concepts have been proposed, no material has so far shown wide-ranging icephobic features, enabling durability and manufacturing on large scales. Herein, we present gradient polymers made of 1,3,5,7-tetravinyl-1,3,5,7-tetramethylcyclotetrasiloxane (V_4_D_4_) and 1*H*,1*H*,2*H*,2*H*-perfluorodecyl acrylate (PFDA) deposited in one step via initiated chemical vapor deposition (iCVD) as an effective coating to mitigate ice accretion and reduce ice adhesion. The gradient structures easily overcome adhesion, stability, and durability issues of traditional fluorinated coatings. The coatings show promising icephobic performance by reducing ice adhesion, depressing the freezing point, delaying drop freezing, and inhibiting ice nucleation and frost propagation. Icephobicity correlates with surface energy discontinuities at the surface plane resulting from the random orientation of the fluorinated groups of PFDA, as confirmed by grazing-incidence X-ray diffraction measurements. The icephobicity could be further improved by tuning the surface crystallinity rather than surface wetting, as samples with random crystal orientation show the lowest ice adhesion despite high contact angle hysteresis. The iCVD-manufactured coatings show promising results, indicating the potential for ice control on larger scales and various applications.

## Introduction

The presence of ice in transportation systems, such as aircraft^1^ and infrastructure, such as bridges,^2^ building-integrated photovoltaics,^3,4^ communication towers,^5^ power lines,^6^ wind turbines,^7,8^ or roads, may lead to severe malfunction, which not only interrupts the service but potentially endangers life. Although the system design contemplates withstanding different environmental conditions, ice formation and accretion still represent a critical issue. Currently, active anti-icing and deicing technologies (e.g., heating systems^9^) are employed to attenuate icing problems. Unfortunately, these systems rely on a constant external power supply and perform mainly once ice has formed. Moreover, they consist of multicomponent systems that require constant design optimization to operate in different environmental conditions and applications. This has naturally limited their feasibility as a long-term solution. In the last years, attention has turned toward the development of passive anti-icing systems.^10^ This approach relies on materials intended to be permanently incorporated on surfaces of interest and prevent ice formation by inherently inhibiting the ice nucleation, delaying the ice formation, and/or reducing ice adhesion without any external power input. Materials possessing one or several of these attributes are referred to as icephobic.

Different conceptual frameworks have been used in the development of icephobic materials.^11−17^ Within the proposed concepts, superhydrophobicity is the most common working principle for designing novel anti-icing coatings.^18−20^ Superhydrophobic surfaces are tailored to promote the shedding of water by drop rebound or aerodynamic drag; thus, water can incorporate into the external flow, significantly reducing the probability of the water freezing on the surface.^21^ Yet, several reports have confirmed that even efficient superhydrophobic surfaces do not necessarily perform as feasible icephobic surfaces.^22^ The icephobic performance of superhydrophobic textured surfaces is highly questionable since changes in environmental conditions, such as humidity, can cause the textured surface to serve as a highly effective interlocking site during ice formation.^13,23^ Icephobic properties are not only related to the inherent properties of the material but also to the environmental conditions and the type of ice formed. These external variables are the greatest challenge of icephobic materials.^24,25^ Industrial applications require robust materials that can withstand harsh environments; therefore, durability is crucial for the implementation of such materials. Furthermore, the manufacturing requires to be adequate to successfully functionalize larger areas, several shapes, and different materials.

Initiated chemical vapor deposition (iCVD) is a powerful one-step technique in the production of thin films from vapor precursors based on free radical polymerization.^26^ Over the last few years, iCVD has been applied in electronics,^27,28^ biomaterials,^29,30^ pharmaceuticals,^31^ and sensors,^32−35^ establishing itself as a powerful process. Upon the emerging demand for new green policies and social attempt to shift toward environmentally friendly processes, iCVD presents an excellent solution. This technique reduces to zero the need for solvents during the process and the production of waste. In this regard, it overcomes all conventional wet-chemistry processes with higher precision and uniform coverage, and due to the mild temperatures, nearly any substrate can be coated.^36^

iCVD offers a valuable opportunity for anti-icing applications because the process can be adapted to coat large areas,^37^ different materials, and shapes,^38^ and, at the same time, maintain the optical appearance of the original substrates (e.g., transparency^39^). The scarce literature available for anti-icing coatings produced with iCVD suggests that this approach has not been fully explored. Previous reports by Gleason′s group showed promising results for passive icephobic coatings done with iCVD. It was shown that fluorinated compounds are easily processed with iCVD.^40^ Sojoudi et al.^41^ reported a bilayer structure consisting of 1*H*,1*H*,2*H*,2*H*-perfluorodecyl acrylate (PFDA) and divinylbenzene (DVB). However, this approach required a pretreatment of the substrate with solvents plus plasma and intermediate steps during the deposition.

Schröder et al.^42^ suggested the potential of gradient polymers for anti-icing coatings. Gradient polymers can be described as structures that exhibit progressive conversion from species A to species B, resulting in a vertical structure consisting of two homopolymer-like sections on each end and a copolymer in between. These structures enable the compatibility of monomers of contrasting natures and allow the full retention of the functional group properties. As discussed elsewhere,^43^ the composition of gradient polymers can be freely tailored with high precision over the entire thickness of the coating, resulting in independent properties at each section of the structure. The creation of these robust structures is, so far, exclusive of vapor deposition techniques. In Schröder’s work, the mechanical and chemical advantages of a gradient polymer structure containing 1,3,5-trivinyl-1,3,5-trimethylcyclotrisiloxane (V_3_D_3_) in the bottom section and poly(tetrafluoroethylene) (PTFE) in the top section were shown. However, the icephobic properties of the material were not assessed.

In the study by Huang et al.,^44^ an effective coating that delayed frost formation and icing was achieved through a polymeric nanoarray using only iCVD. By bringing the PFDA monomer to its condensation point inside the reactor, nanodrops formed over the substrate, and from there, nanocone arrays grew by a “vapor–liquid–solid” mechanism. The combination of a nanotextured surface and a low surface energy obtained by the fluorinated compound granted the ability to entrap air that reduced the heat conduction and the contact area between the coating and the drops. Furthermore, the very low surface energy obtained hindered the condensation of water vapor and slowed the growth of the condensed drops. However, nothing is reported about ice adhesion performance. It is very unlikely that the ice adhesion strength can be reduced on textured surfaces due to physical interlocking as previously observed and reported.

In this study, we combined the strongest attributes of the approaches reported so far using iCVD to introduce gradient polymers as an effective alternative to mitigate ice formation and accumulation. A gradient polymer with a bottom section with 1,3,5,7-tetravinyl-1,3,5,7-tetramethylcyclotetrasiloxane (V_4_D_4_) and a top section with PFDA was constructed in one step using iCVD. The choice of using PFDA as a fluorinated monomer was also dictated by the strong crystalline aggregation that its polymer shows.^45^ In addition, depending on the iCVD deposition conditions, it is possible to tune the orientation of the crystallites with direct consequences on the wettability.^46^ We tested the icephobic properties of these gradient polymers intending to obtain all desired properties for an icephobic surface: delayed ice nucleation and freezing time, depressed freezing temperature, reduced ice adhesion, and at the same time, extraordinary durability and adhesion. Contrary to the predominant concept of creating high-repellency surfaces to reduce the contact time and thus avoid ice nucleation and frost formation, we show that strong pinning of the microcondensed drops can lead to an effective delay of ice nucleation and a better control of its propagation. This approach is more effective than avoiding the inevitable formation of frost and ice.

## Experimental Methodology

### Materials and Deposition Process

A series of gradient polymers with different properties were synthesized and deposited using a custom-made iCVD reactor with a standard configuration described elsewhere.^26^ Di-*tert*-butyl peroxide (TBPO) was purchased by Sigma-Aldrich and used with no further purification as an initiator. 2,4,6,8-Tetraethenyl-2,4,6,8-tetramethylcyclotetrasiloxane (V_4_D_4_) and 1*H*,1*H*,2*H*,2*H*-perfluorodecyl acrylate (PFDA) were both purchased by Sigma-Aldrich and used with no further purification as monomers. The iCVD reactor was operated in a continuous flow mode. The monomers V_4_D_4_ and PFDA were heated to ensure a constant flow into the reactor up to 80 and 95 °C, respectively. The flow rates for TBPO were 1.0 ± 0.1 sccm, for V_4_D_4_, those were 0.2 ± 0.05 sccm, and for PFDA, those were 0.2 ± 0.05 sccm. The flow rates were controlled using a needle valve. The reactor was operated at a pressure of 500 mTorr with a filament temperature of ∼200 °C and a substrate temperature of 40 °C.

The deposition thickness was followed in situ using a He–Ne laser using silicon substrates as reference. For the formation of the gradient polymer first, the initiator and V_4_D_4_ monomer were introduced into the reactor. The monomer V_4_D_4_ was used to form the bottom section of the gradient polymer until the desired thickness was achieved. Then, the monomer PFDA was gradually introduced into the reactor. When the desired thickness of the copolymer p(V_4_D_4_-*co*-PFDA) was reached, the monomer V_4_D_4_ was gradually stopped. Hence, the top section was formed only from the PFDA monomer. The thicknesses of the different sections were: 50 nm for the bottom pV_4_D_4_, 150 nm for the p(V_4_D_4_-*co*-PFDA), and 100, 200, and 300 nm for the top pPFDA sections. The gradient polymers so built were named Grad100, Grad200, and Grad300 depending on the thickness of the top section. For comparison, a sample with stacked layers was produced, in which there was no copolymerization and 100 nm of pPFDA was deposited on 50 nm of pV_4_D_4_ with a sharp interface. The coatings were deposited on silicon substrates.

### Characterization

The coating thickness was determined via ellipsometry (M-2000 V ellipsometer from J.A Woolam Co). Static, advancing, and receding water contact angle measurements were conducted using 10 μL of deionized water and advancing–receding rates of 0.4 μL/s. Roll-off angle measurements were performed with drops of 10, 15, and 20 μL. A Biolin Scientific’s Optical Tensiometer Theta Flow was used. Fourier transform infrared (FTIR) analysis was performed under vacuum using a Bruker IFS 66 V spectrometer. Atomic force microscopy (AFM) analysis was performed with a Nanosurf Easyscan 2 AFM with a scanning probe model PPP-NCLR-20 in tapping mode. Cross-hatch adhesion test was used to assess the adhesion strength of the coating in the substrates, the methodology ASTM D3359, method B was selected as the most suitable for the coatings. In this method, 11 patterned cuts along the sample with another 11 patterned cuts perpendicular to the first resulted in a squared mesh. The ASTM pressure-sensitive tape was applied and then removed. The adhesion of the coating was evaluated by comparing the damage with standardized images and descriptions. The atomic composition of the polymers was determined by X-ray photoelectron spectroscopy (XPS). The spectra were acquired using non-monochromatic Mg Kα radiation (1253 eV). The pass energy was 50 eV for survey scans and 20 eV for high-resolution scans. The takeoff angle was 55°. The analysis of the data was performed using Casa XPS. Grazing-incidence X-ray diffraction (GIXD) measurements were performed at the synchrotron Elettra XRD1 beamline in Trieste, Italy. The primary X-ray beam had a wavelength of 1.4 Å. To detect the diffracted beam, a Pilatus 2 M detector placed 200 mm from the sample was used. The experimental data were transformed into reciprocal space through the utilization of GIDVis software.^47^

### Frost Nucleation

To observe the mechanism and evolution of ice nucleation from water vapor condensation, the substrates were placed over a cooling stage and the temperature was decreased from room temperature to −20 °C, maintained for as long as the observable area was fully covered with frost, and then heated to the initial temperature. A Linkam horizontal heatcell stage M-2000 (minimum temperature of −196 °C, temperature accuracy and resolution of 0.01 °C, and temperature stability of 0.05 °C) was coupled with a Leica Wild M3B microscope. The relative humidity (RH ∼ 50%) was constant throughout the experiments. The process was recorded and then analyzed using the software ImageJ (a schematic representation of the setup is shown in the Supporting Information, Figure S1a).

### Drop Freezing Delay

Deionized water drops of 10 μL were placed over the substrates, and the temperature was decreased from room temperature to −20, −25 and −30 °C using the Linkam stage described in the previous section. The temperature was maintained constant until the drops froze. These events were identified and recorded with high precision using an infrared camera Optris model PI160. A sudden release of heat corresponding to the recalescence stage in the freezing process indicates the beginning of the change of state from liquid to solid.^48^ These experiments were repeated at different relative humidities (<10, ∼50, and >70%). To assess the effect of the cooling rate on the drops freezing, a 10 μL drop was placed on the substrate and the temperature was decreased several times in continuous cycles from room temperature to −20 °C at different cooling rates from 10 to 50 °C/min (a schematic representation of the setup is shown in the Supporting Information, Figure S1b).

### Freezing Point Depression

The freezing point of water placed on the gradient polymers was determined by in situ X-ray diffraction during freezing. A low-temperature chamber allowed us to measure the exact temperature of the crystallographic transition of water when the drop froze. The cooling rate was 1 °C/min from 10 to −25 °C. The experiments were done in an air atmosphere and repeated under a N_2_ atmosphere. An Anton Paar’s XRDynamic 500 equipped with a TTK 600 low-temperature chamber with liquid nitrogen cooling was used. It was equipped with a Primux 3000 sealed-tube X-ray source with the Cu anode and a Pixos 2000 detection unit featuring a solid-state pixel detector was used. A divergent beam Kα1,2 monochromator was placed in the primary beam path to work in an optimized Bragg–Brentano geometry.

### Ice Adhesion

To quantify the strength in which ice adheres to different substrates, a custom-built setup measuring the ice adhesion force was used. This setup was built at the University of Milano-Bicocca and described elsewhere in detail.^49^ Cylindrical molds with inner diameters of 8, 10, 12, and 14 mm were filled with distilled water, placed over the substrates, and frozen at −15 °C. A metallic rod coupled to a force gauge pushed the mold at a constant velocity (0.01 mm/s) and measured the force at which the ice was detached. To avoid ambient condensation, the relative humidity is decreased through a continuous nitrogen supply inserted into the chamber (ambient temperature *T*_amb_ = 20 °C, RH < 3%). To classify the ice detachment mechanism, the detachment was recorded using a high-speed camera (PHOTRON NOVA FASTCAM S6, Venus Laowa 100 mm *f*/2.8 2× Ultra Macro APO lens, JJC Auto Focus Extension Tube 20 mmm).

## Results and Discussion

**Figure 1:**
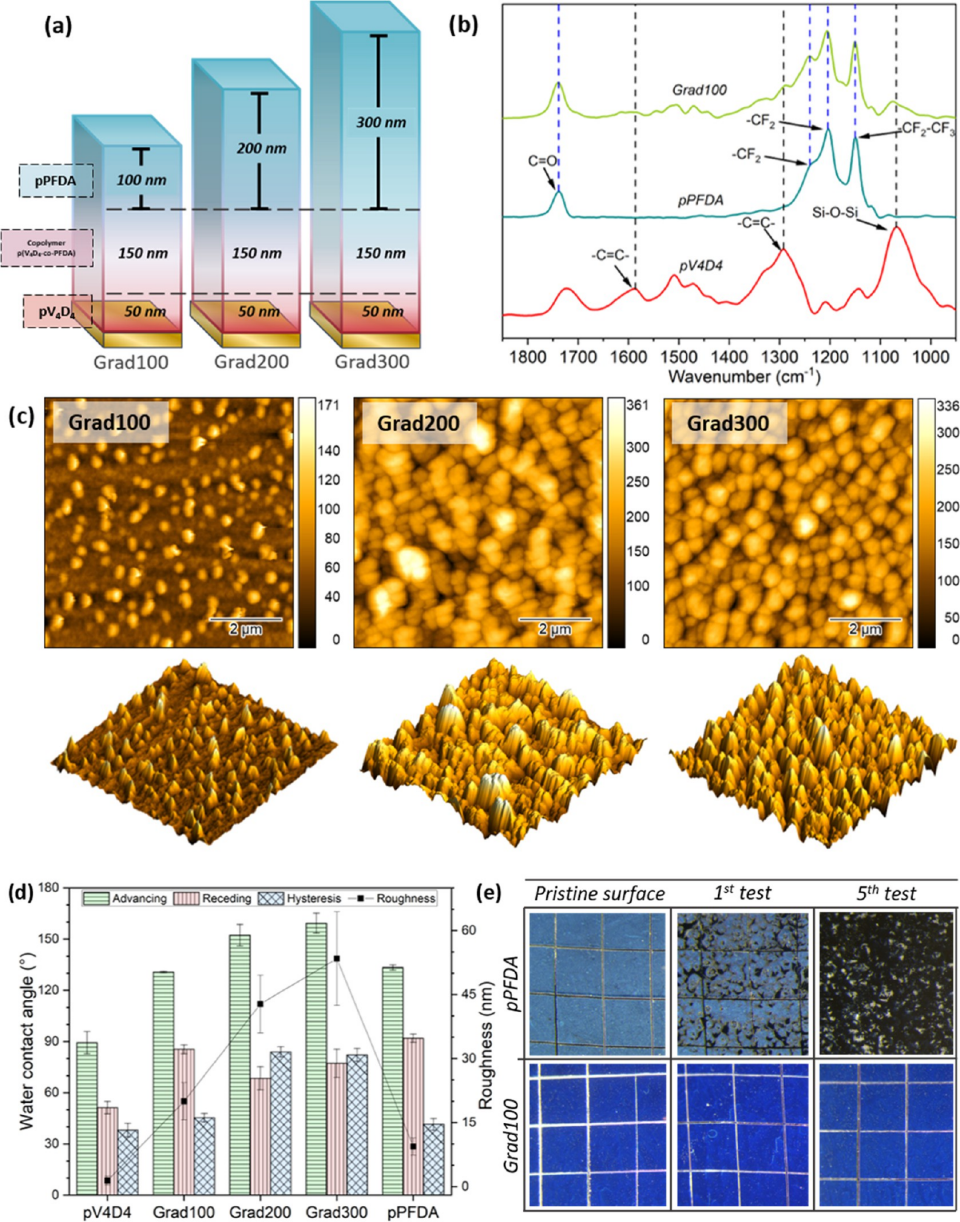
(a) Schematic representation of the structure of a gradient polymer, where no interphases are distinguished. (b) The FTIR spectra of pV_4_D_4_, pPFDA, and a gradient polymer show that the functional groups of interest are retained after the polymerization and deposition. (c) Atomic force micrograph of the gradient polymer surface in 2D and 3D. Through the 3D surface representation, the systematic increment in roughness is evidenced. (d) Comparison of the advancing, receding, WCA hysteresis, and roughness of the different sections that constitute the gradient polymer and the different top section thicknesses. (e) Optical microscopy images of the sample before and after the cross-hatch adhesion test. No damage was visible on the gradient surfaces after the tests (pV_4_D_4_, Grad100, and Grad200 can be found in the Supporting Information, Figure S3).

Vertical gradient thin-film polymers were deposited via iCVD, resulting in a coating structure schematically represented in Figure 1a. Gradient polymers are structures with a progressive transition from one species to another without sharp interfaces. Three sections with different properties can be distinguished within the structure: the bottom section of the gradient polymer, which is in direct contact with the substrate, consists of a V_4_D_4_ homopolymer section. This monomer forms highly cross-linked networks due to the four available vinyl groups present in its molecular structure. The cross-linked network provides mechanical stability to the structure because the networks can adhere with high strength to a rigid substrate. As previously described,^42^ compatibility in stiffness is the key to good adhesion that results in high bond strengths. The middle section is formed from the moment when the monomer PFDA was gradually introduced into the reactor. This leads to the gradual formation of a copolymer p(V_4_D_4_-*co*-PFDA) that starts with a rich V_4_D_4_ part and ends with a part rich in PFDA units. The copolymer acts as a “bridge” in favor of a subtle transition between two sections that possess different chemical and physical properties, hence avoiding a sharp interface. The top part is formed above the copolymer, when the V_4_D_4_ flow is completely stopped, leading to the PFDA monomer units forming a PFDA homopolymer section at the top. Gradient polymers with 100, 200, and 300 nm top PFDA sections (referred from here as Grad100, Grad200, and Grad300, respectively) were deposited to study the effect on the coating surface properties.

The infrared spectra shown in Figure 1b confirmed a successful synthesis: the main characteristic signals of the homopolymers pPFDA and pV_4_D_4_ were present in the gradient polymers, indicating the successful retention of the groups after polymerization and deposition. The analysis is shown in the Supporting Information, Section 1. X-ray photoelectron spectroscopy (XPS) measurements revealed that there was no significant difference in the elemental composition among the different gradient polymers and pure pPFDA, meaning that the gradient polymer top sections have a chemical nature as pure as the homopolymer pPFDA. Data is shown in the Supporting Information, Section 2.

Atomic force microscopy (AFM) imaging (Figure 1c) revealed that the surfaces possessed features with different shapes, sizes, and distributions leading to different properties. The pPFDA showed a nanoscale roughness (RMS = 8 ± 1 nm), in which tiny and very dispersedly distributed aggregates were observed (Figure S2). These aggregates are typical of a pPFDA island growth.^45^ On the other hand, the gradient polymers exhibited spherical aggregates, and as the top section increased, they got more densely populated, resulting in a significant increase in roughness, RMS = 20 ± 4, 43 ± 6, and 53 ± 10 nm for Grad100, Grad200, and Grad300, respectively. The differences in topography arise from the underlayer on which the pPFDA section grows: the homopolymer grew upon a silicon substrate, a flat and spotless surface, whereas in the gradient polymer, the top section grew from the copolymer surface, which is composed of islands with an irregular shape and higher roughness than silicon and pV_4_D_4_ (Figure S2). Those inhomogeneities induced nucleation points from which the PFDA growth resulted in spherical aggregates observed in the gradient polymers.

The difference in morphology affected the surface wettability. As shown in Figure 1d, the gradient polymer surfaces displayed a hydrophobic behavior, an apparent increment in hydrophobicity can be observed when the top section increases. Surprisingly, contact angle hysteresis consisted of high values, 45° ± 2°, 83° ± 2°, and 82° ± 3° for Grad100, Grad200, and Grad300, respectively, as confirmed by low drop mobility: the roll-off angle for all of the gradient polymers exceeded 90° with 10 and 15 μL drops, and the samples could even be flipped 180° with the drops staying still. Only for 20 μL drops, the gravity forces overcome capillary adhesion forces and drops slide.

The cross-hatch adhesion test was employed to determine the coating adhesion to the substrate. The ASTM D3359 standard was used, and the samples were examined with a microscope before and after the adhesive tape was removed from the coating. The procedure was repeated 5 times over the same sample and the same region. Figure 1e shows the comparison of pPFDA with Grad100. The coating showed a clean and homogeneous surface before adhering to the tape, in which the lattice pattern was clearly visible. After adhering and removing the tape for the first time, the homopolymer pPFDA coating showed evident damage; after the fifth test, the coating was almost completely removed. The gradient polymers exhibited an extraordinary adhesion to the substrate and no evidence of damage was observed after the fifth test within the squares or at the edges where the substrate surface was exposed. It is outstanding that the adhesion of nanometric coatings could be examined using this method, designed mainly for thicker and tougher materials. This evaluation highlighted how the gradient structure successfully overcomes the intrinsic low stability and poor adhesion of the fluorinated coatings without losing its desirable functionality.

**Figure 2:**
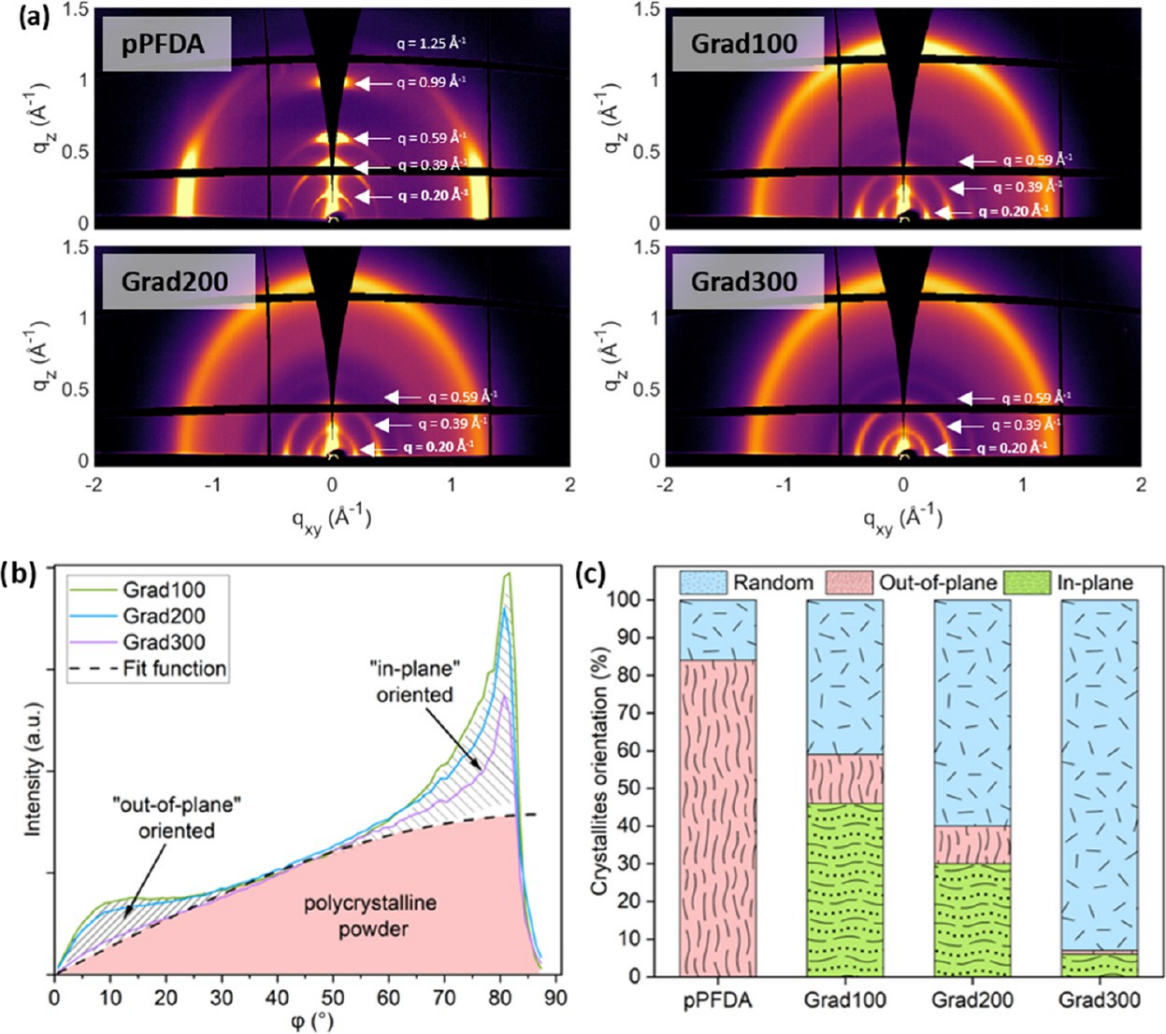
(a) GIXD maps of pPFDA and gradient polymer samples. The prominent signal observed along the *q*_*z*_-axis in pPFDA indicates the orientation of crystal structures “out-of-plane”. The intensity signal at the *q*_*xy*_-axis and the arc, as observed in the gradient samples, indicate “in-plane” and random orientation, respectively. (b) Comparison of the corrected intensity profile (at *q* = 0.20 Å^–1^) of gradient polymers. (c) Crystallite orientation fraction of the pPFDA and gradient polymers.

The structural analysis of the gradient polymers was done through grazing-incidence X-ray diffraction (GIXD) measurements. The GIXD maps of pPFDA and the gradient polymers are shown in Figure 2a. The diffraction peak in the homopolymer pPFDA at the scattering vector *q*_*z*_ = 0.20 Å^–1^ corresponds to a bilayer lamella structure formed when the PFDA pendant fluorinated groups of two chains align “face-to-face” after polymerization. This structure is known as the smectic B phase. The Bragg peak at *q*_*z*_ = 0.39 Å^–1^ is the second-order peak and corresponds to a single pendant group. The dimensions of the double and single pendant groups correspond to *d* = 32.4 Å and *d* = 16.2 Å, respectively. Peaks at *q*_*z*_ = 0.59 Å^–1^ and *q*_*z*_ = 0.99 Å^–1^ are the third- and fifth-order diffraction peaks, respectively.^45,50,51^ In pPFDA, these signals appeared strong along the *z*-axis, indicating an “out-of-plane” orientation of the crystallites, meaning that the polymer backbone chains aligned parallel to the substrate and with the fluorinated lamellas aligned perpendicular to the substrate surface. The small arc-like shape revealed a rather low mosaicity in the pPFDA sample.

In the gradient polymer samples, the diffraction peaks are seen at the same |*q̅*| values as for pPFDA, but the diffraction intensity is rather homogeneously distributed along the Debye–Scherrer rings, resulting in a three-dimensional (3D) powder-like or random texture. The signal differences from the homopolymer pPFDA indicated that different crystallite orientations can be induced whether pPFDA is deposited on Si or grows on a different surface, as in the case of the gradient polymers, that grow above the copolymer p(V_4_D_4_-*co*-PFDA). The signals at the q_*xy*_-axis indicate an “in-plane” orientation of the crystallites, which refer to the backbone chains aligned perpendicular to the substrate with the lamellas parallel to the substrate. The “in-plane” orientation directly exposes the fluorinated CF_2_ and CF_3_ groups at the surface. This orientation produces intrinsically an extremely low-energy surface compared to the “out-of-plane” orientation, in which the CH_2_ chains are exposed at the surface. As reported, fluorinated compounds are the solids with the lowest possible energy surfaces due to their very low dielectric properties.^52^

From the GIXD analysis, it was possible to estimate the crystallite fraction at different orientations. For this analysis, the intensity profile along the polar angle of the scattering signal at |*q̅*|= 0.20 Å^–1^, which corresponds to the first-order peak, was taken as a reference. Although this peak occurred at low q-values and close to the incoming beam, it was used because of the experimental inaccessible area (missing wedge) of the other peaks. The intensity profiles were extracted from the two-dimensional (2D) maps and later corrected by applying a Lorentz correction factor (sin(ϕ)). This correction is valid if the crystallites have an isotropic orientation with respect to the surface normal, which both the 2D and 3D powder textures possess.^53^

Figure 2b shows the comparison of the corrected intensity profiles of the gradient polymers for each angle (ϕ) of the arc (i.e., from 0 to 90°). The three distinguished areas correspond to the estimated fraction of crystallites at different orientations. The quantitative estimation is shown in Figure 2c, where the pure pPFDA composition is estimated to be predominantly formed by the crystallites oriented “out-of-plane” (84%) and the rest by randomly orientated. As the gradient polymers were formed, two main things were observed: a strong shift toward randomly oriented structures and the appearance of “in-plane” crystallites, naturally, at the expense of crystallites oriented “out-of-plane”. Grad100 consisted of 41% of crystallites randomly oriented, 46% of crystallites “in-plane”, and 13% of crystallites “out-of-plane”. Grad200 consisted of 60% of crystallites randomly oriented, 30% of crystallites “in-plane”, and 10% of crystallites “out-of-plane”. Grad300 consisted of 93% of crystallites randomly oriented, 6% of crystallites “in-plane”, and 1% of crystallites “out-of-plane”: as such, a progressive disappearance of the “out-of-plane” orientation and the transformation toward a more randomly oriented structure is observed.

In a randomly oriented configuration, “in-plane” and “out-of-plane” orientations coexist. In other words, in gradient polymers, regions with the carbon backbone exposed to the surfaces and regions with fluorinated groups exposed are present on the same plane. The surface energy is quite contrasting between these two regions, resulting in a surface energy discontinuity that is larger as the top section thickness increases. We believe that this discontinuity, together with the change in surface roughness, caused the difference in wettability from the pure pPFDA and within the gradient polymers. The discontinuity causes different surface properties because of the different molecular interactions occurring between the coating and water molecules.

The gradient polymer architecture provided adhesion and stability and simultaneously enhanced the surface properties. Since the change of thickness is a straightforward controllable parameter, the surface properties of these materials can be easily tuned. Previous studies showed how the orientation of lamellas in pPFDA was regulated by iCVD deposition parameters, such as filament temperature, substrate temperature, or initiator–monomer flow rate.^45,50^ However, no studies have reported inducing certain orientations of the crystallites through polymeric architecture. This crystallographic analysis was the last piece in the puzzle of understanding the origin of the differences between surfaces with identical chemistry. It will be discussed how and why this has a remarkable impact on the icephobic properties of the material.

### Icephobic Performance of Gradient Polymers

#### Ice Adhesion

Among the different icephobic attributes, reducing ice adhesion is probably the most relevant and desired for general applications of icephobic surfaces. According to the literature, passive icephobic coatings should reduce ice adhesion strength, τ_ice_, calculated as the ratio between the force F that needs to be applied to remove a piece of ice from the surface and the iced area A; F/A. Values below 100 kPa would guarantee an ice detachment with low external load.^54^ The value τ_ice_, however, is a source of discrepancies as the experimentally measured average ice adhesion strength τ_ice_ is not only a material property but it also depends on many factors, such as test system parameters and detachment mechanism. As indicated by Golovin et al.,^55^ the definition of τ_ice_ implies that the force scales with the iced area, which is not always the case. Specifically, *F*/*A* is found constant in the stress-dominated regime for small ice length scales but not in the toughness-dominated regime for ice dimensions higher than a critical length *L*_c_. For this reason, in this work, we first identified the fracture mechanism and then described the ice removal performance with the proper quantification.

**Figure 3:**
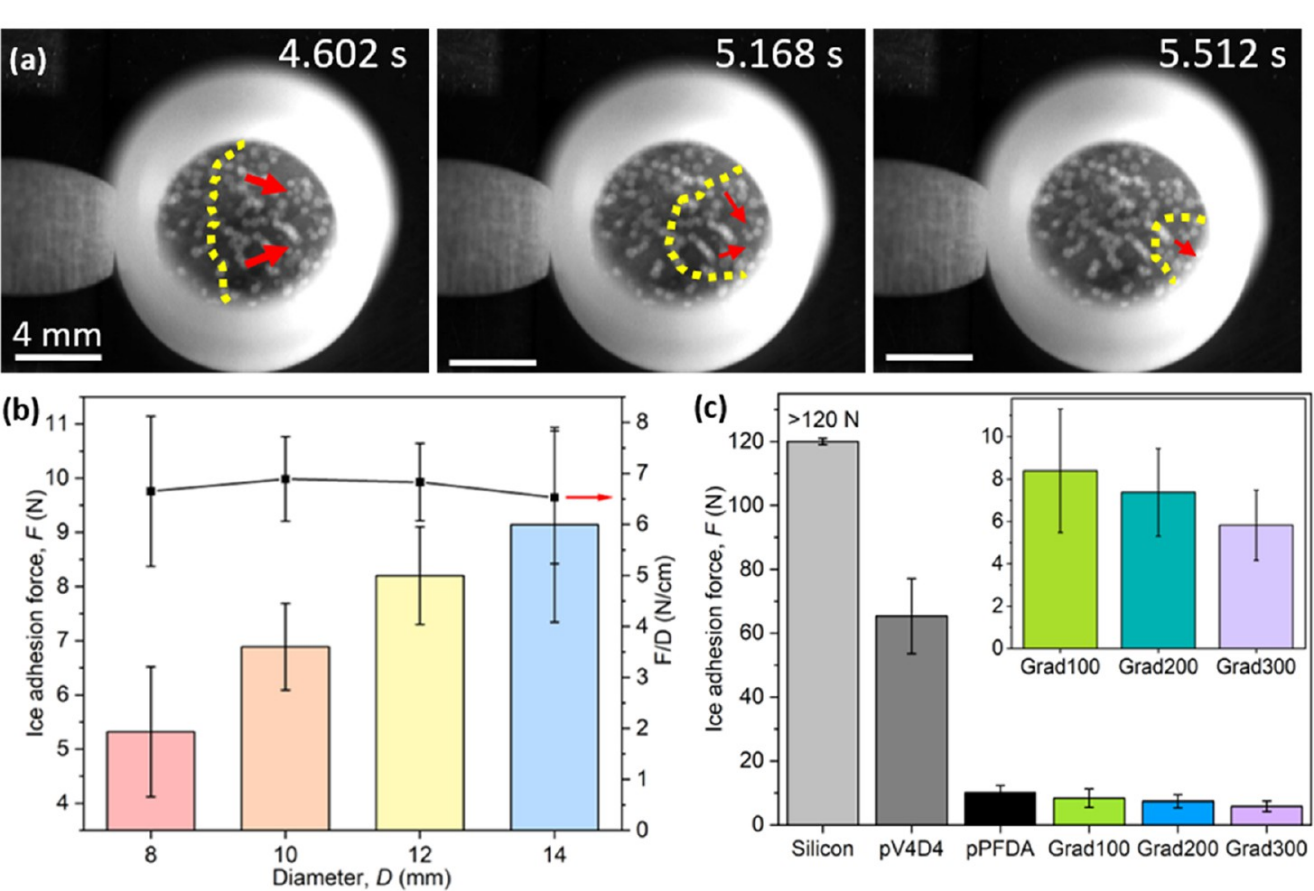
(a) Snapshots of the crack propagating through ice, and this indicated a toughness-dominated detachment. (b) Ice adhesion force and the force per unit diameter for Grad300 as a function of ice mold diameter. (c) Ice adhesion force for several coatings and gradient polymers are shown in detail in the inset. As the top section thickness increased, the ice adhesion force decreased. Values for the pPFDA coating were consistent with the literature.^41^ Grad300 exhibited an adhesion reduction factor (ARF) of at least 20 times compared to the silicon substrate.

Figure 3a shows three representative frames from a high-speed video taken during an ice detachment experiment, in which a mold of ice of diameter *D* was pushed on the surface of the Grad300 coating (see the full sequence in the Supporting information, Video S1). The frames show a clear fracture propagation, indicating that the ice detachment follows a toughness-dominated mechanism.^56,57^ This represents an extraordinary case since, at such small ice length scales, an instantaneous failure due to stress-dominated detachment is more commonly observed. This was validated by repeating the experiments using molds of different diameters, as shown in Figure 3b: it was found that the ratio *F*/*D* is constant, and not *F*/*A*, which would be true for stress-dominated detachment. Since in our experiments, it was only possible to detach the ice column via a toughness-dominated mechanism, the critical length (defined as the smallest length at which the toughness-dominated regime is observed) is lower than 0.8 cm. Such a low critical length value has never been reported before. Hence, gradient polymers are classified as “low-interfacial toughness” (LIT) materials, where the force required to detach ice is not only low but also independent of the interfacial area.^58^

Figure 3c shows the ice adhesion force of the gradient polymers compared to silicon, pV_4_D_4_, and pPFDA. The gradient polymers show the lowest ice adhesion. Moreover, the trend shows how icephobicity enhanced as the top section thickness systematically increased, contrary to other LITs that have demonstrated to perform better by minimizing the thickness.^55^ Very noteworthy is the comparison of the ice adhesion force between pPFDA and Grad300. These two films had the same total thickness (500 nm); nevertheless, their adhesion force is quite different: 10 vs 6 N, respectively. No surface chemical differences existed in both samples and the contact angle hysteresis was significantly higher on Grad300 than on pPFDA (see Figure 1d discussed above), suggesting that the difference in icephobicity between the two samples cannot be described in terms of hydrophobicity. The only material property between these two surfaces that could explain the ice adhesion reduction was the difference in crystal orientation. In particular, the crystal random orientation on Grad300 appears to be responsible for the lowest ice adhesion.

In addition, ice detachment experiments served as an indirect method to assess the durability of the coating and its resistance against scratches. Gradient polymer coatings showed an enduring performance with no damage, whereas pV_4_D_4_ and pPFDA coatings were severely damaged after multiple repetitions of the experiments. Furthermore, to provide an all-around characterization of the coating’s behavior in icing conditions, we have conducted freezing delay, freezing point depression, condensation, and frost growth tests, which are presented in the following sections.

### Drop Freezing Delay

**Figure 4:**
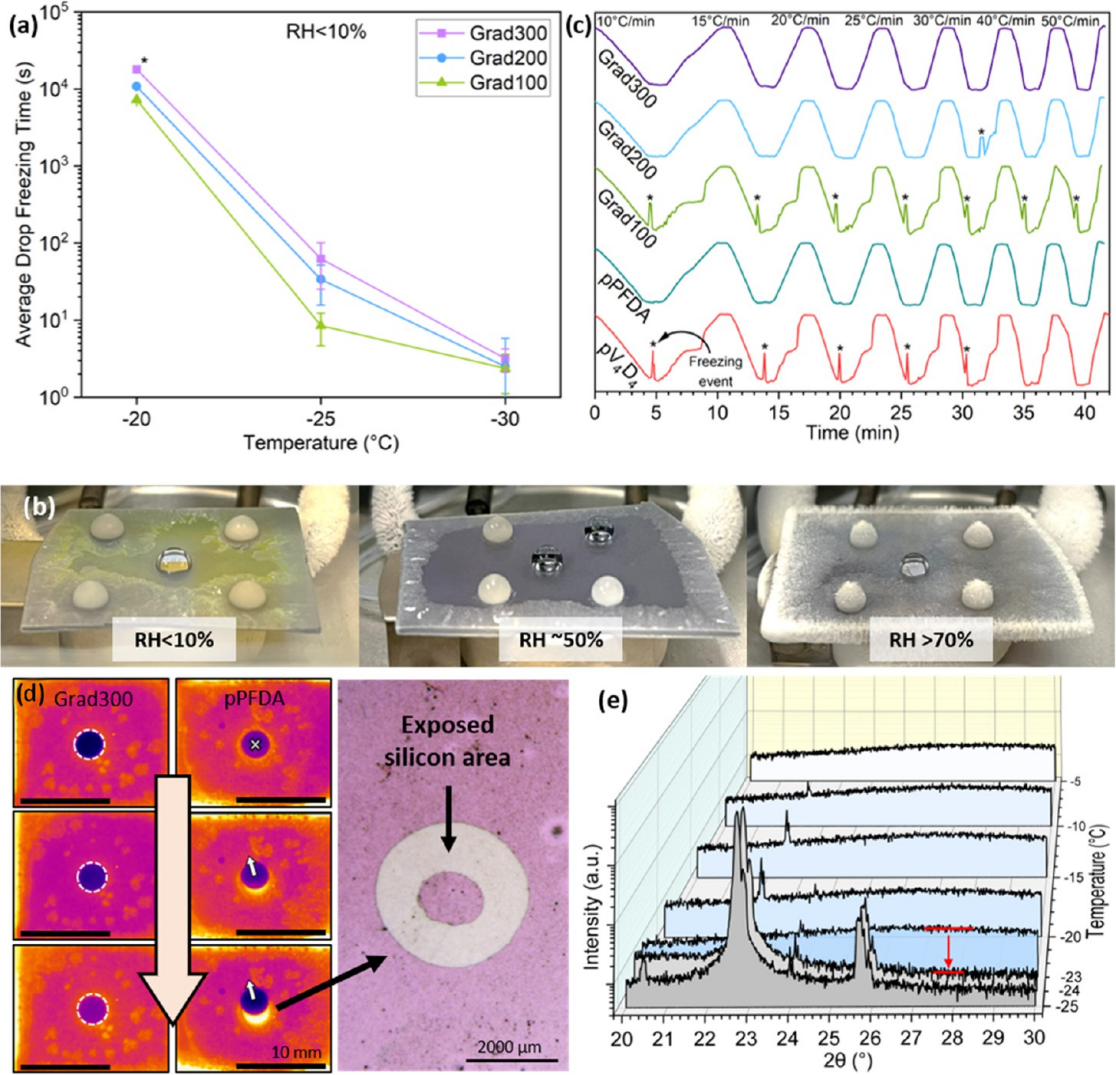
(a) Drop freezing time is plotted as a function of temperature, where the exponential temperature dependency follows the classical nucleation theory, (*) indicates the experiments limited to 5 h. (b) During the drop freezing delay experiments, different types of frost developed at different relative humidities. At low relative humidities (<10%), it grew in a dendritic mode along the surface; at medium relative humidities (∼50%), the frost grew similarly close to the surface but noticeably thicker; at high humidities (>70%), a voluminous frost with dendrites growing mainly out-of-plane was observed. (c) Cooling cycles at different cooling rates, in which the probability of drop freezing was significantly reduced in the Grad200 and Grad300 surfaces. (d) Infrared snapshots during the deicing of the frozen drop in the Grad300 and pPFDA substrate. In Grad300, the drop remains static. In pPFDA, the drop is displaced from its original position tearing the coating and exposing the silicon substrate. A microscopy image reveals the damaged region, where the pPFDA coating was torn. (e) In situ XRD diffractogram at continuous temperatures from −5 to −25 °C. The freezing occurred at −24 °C (in Grad200). The diffractogram in 3D shows clearly the transition from an amorphous state (−23 °C) to a crystalline state (−24 °C) by the flattening of the curve (indicated by the red arrow) and the appearance of the characteristic peaks.

Different trends were distinguished from these experiments, similar to those previously reported.^59^ The drop freezing time increases exponentially as a function of temperature, regardless of the relative humidity for all samples. Nonetheless, a systematic increment in the freezing delay from Grad100 to Grad300 was observed (Figure 4a). During the experiments, two different freezing mechanisms were distinguished according to the temperature. At −15 and −20 °C, the freezing of the drops was mainly triggered by the contact of the frost growing over the surface. Consequently, drops nearer to the edges were the first to freeze. For experiments at −25 and −30 °C, the drops froze spontaneously and the position in which the drops were distributed over the substrate was not relevant. As the relative humidity increased, frost developed differently over the coatings due to a naturally higher concentration of water in the environment (Figure 4b). The gradient polymer surfaces displayed an astonishing performance in delaying the freezing of the drops. Precisely, at low relative humidities, an exact value cannot be provided because the experimental setup is limited to continuously operating for up to 5 h. A summary of the freezing delay can be found in the Supporting Information, Section 5.

The effect of the cooling rate on drop freezing was investigated. A drop was cooled down to −20 °C at seven different cooling rates (10, 15, 20, 25, 30, 40, and 50 °C/min) in cycle, one after the other. In the homopolymer pV_4_D_4_ and Grad100, running continuous cooling cycles triggered the freezing of the drop each time −20 °C was reached. pPFDA, Grad200, and Grad300 showed a high likelihood of suppressing the freezing of the drop independently of the cooling rate (Figure 4c). Drops rarely froze on the pPFDA surfaces but when it happened, the weakest feature of this coating was exposed during the deicing: its durability. As the drop froze, local stresses within the drop due to volume change were strong enough to tear the coating, exposing the substrate. As a consequence, the drop displaced from its original position (Figure 4d). Evidently, pPFDA coatings are not viable for icephobic applications, as the coating durability is low enough to be torn by a single freezing event. Gradient polymer coatings, however, did not show any sign of damage during these experiments even at multiple freezing events.

The freezing point of water over the gradient polymer coatings was analyzed using in situ X-ray diffraction experiments as the temperature gradually decreased. These measurements provided the temperature at which water transitioned from an amorphous (liquid) to a crystalline (solid) state. The transition was explicitly identified in the diffractogram by the presence of the diffraction peaks corresponding to the hexagonal ice structure (*I*_h_) at 22.683, 24.138, and 25.742° (2θ). It was also distinguished by the flattening of the curve as the transition occurred. The gradient polymer samples were compared with a silicon blank. The complete diffractogram series can be found in the Supporting Information in Figure S4. In the silicon surface, the ice diffraction peaks, indicating the freezing point of the drop, were registered at a set temperature of −9 °C. The same characteristic peaks appeared for Grad100, Grad200, and Grad300 at lower temperatures, −22, −24, and −23 °C, respectively (Figure 4e). The results suggested that the coatings were thermally insulating the surface, allowing liquid water to be stable at colder temperatures, at least 14 °C lower compared with silicon. Using in situ XRD was an indirect, noninvasive, and extremely sensitive method to detect the phase change.

### Condensation

**Figure 5:**
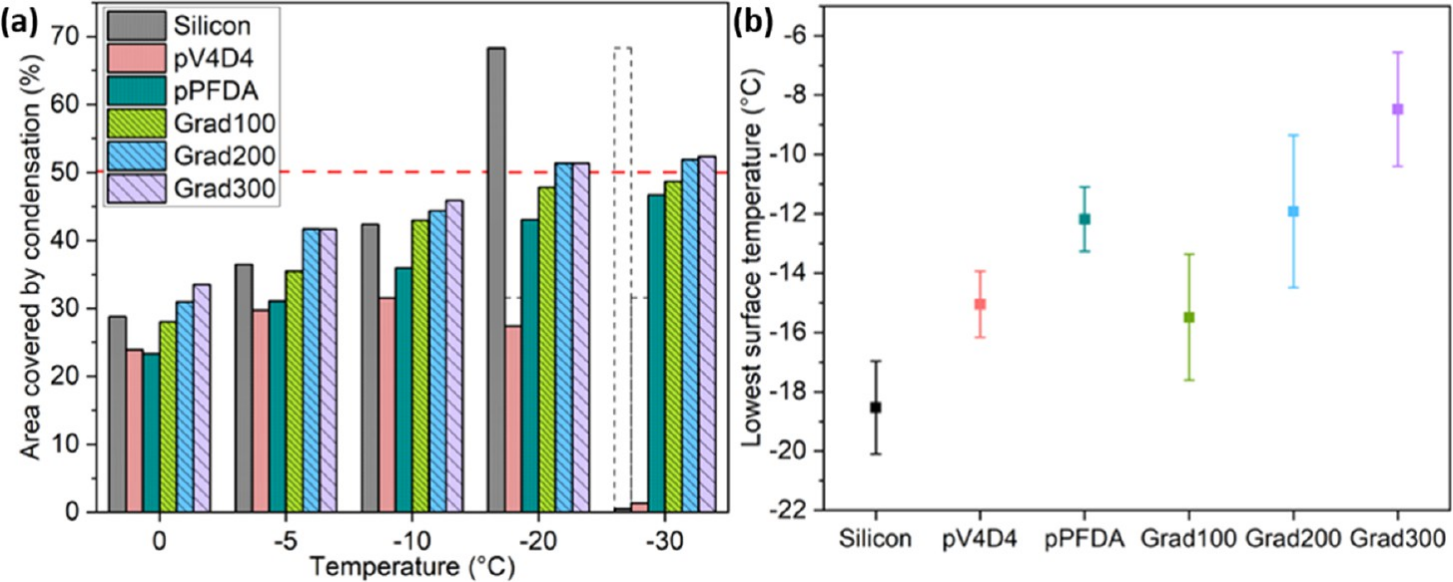
(a) Area covered by condensation at different temperatures. The decrement at −30 °C in the silicon and pV_4_D_4_ samples was due to freezing. The dashed lines indicate the highest area covered reached. (b) The lowest surface average temperature registered at a set point of −20 °C.

As temperature decreases, water from the environment condenses over the surface, the profile and amount of condensed water over the surface highly influence how and when it freezes. Therefore, prior to freezing, condensation was observed and quantified. Figure 5a shows the condensation at different temperatures, quantified as the percentage of area covered by condensed water. Intuitively, as the temperature decreased, condensation increased and a larger area was covered by water. A systematic trend among the gradient polymers was identified: as the top section thickness increased, so did the condensation uptake. Surprisingly, below −20 °C, the condensation occupied more than half of the gradient polymer surfaces with no sign of freezing (consistent with freezing delay results, Figure 4a), contrary to silicon and pV_4_D_4_, in which most of the condensed water froze. The liquid state was identified by the reflection of a microscope lamp on the droplets, whereas the frozen state was identified by the increment in opacity, extinguishing the reflection of the lamp (Supporting Information, Figure S5).

When the experiments were repeated using an infrared camera, the thermal insulating property exhibited by the gradient polymers was confirmed. In these experiments, the temperature at the surface was observed and measured, and the profiles revealed that the coated surfaces displayed a significant offset from the set temperature (−20 °C). Figure 5b shows the average of the lowest surface temperatures recorded on the different coatings: for the silicon sample, the lowest temperature was −18 °C ± 1 °C, whereas for Grad100, Grad200, and Grad300, the temperatures were −15 °C ± 2 °C, −12 °C ± 2 °C, and −8 °C ± 1 °C, respectively. The insulator behavior displayed by the gradient polymers is in part contributing to the stability of liquid water at low temperatures, and consequently the ice nucleation delay in the condensed water.

### Frost Rate and Propagation Mechanism

**Figure 6:**
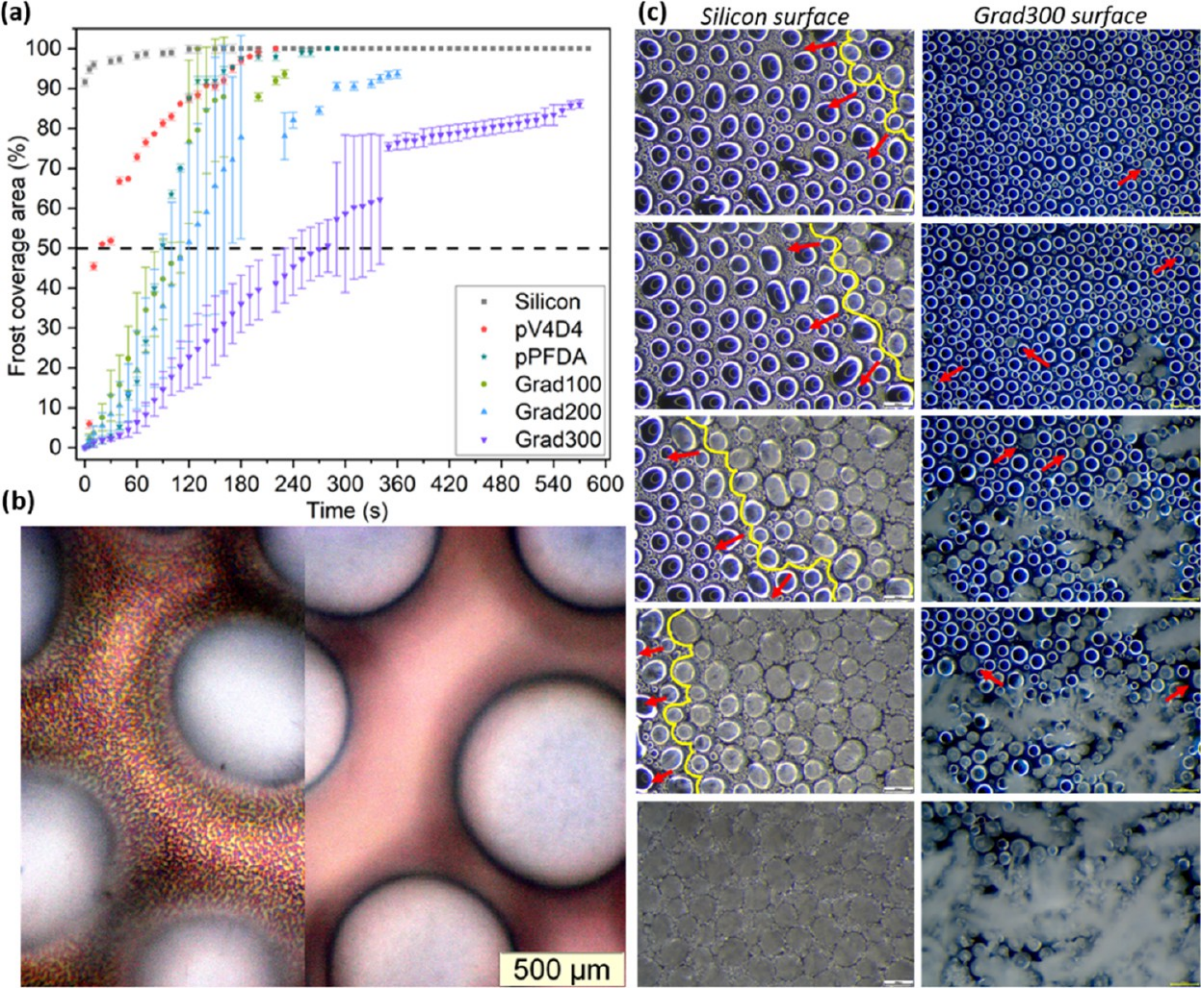
(a) The graph shows the frost coverage area over time of different coatings. (b) A close-up image of droplets formed over the gradient polymer shows the dry zone areas, where the coating is directly exposed to the surface. The left side of the image is focused on the surface. The right side is focused on the longest circumference of the drop. (c) Comparison of microscopy images (observable area of 2.62 mm^2^) revealed the differences in the freezing mechanisms and the resulting frost. In the silicon substrate, the frost front (yellow line) advanced quickly through a chain reaction mechanism, covering instantly the whole area and resulting in a dense and compact ice layer. In gradient polymers, no propagation front was identified; instead, sporadic nucleation points slowly appeared (indicated by red arrows), resulting in a loose and airy frost type. It took 47 times longer to cover the same area.

As the low temperature was maintained, condensed water from the air eventually froze: this type of icing is commonly described as “condensation frosting”.^60^ The temperature was maintained at −20 °C and the development of the condensation frosting was recorded and quantitatively estimated by analyzing the microscopy images using ImageJ. Different condensation frosting mechanisms were distinguished, crucial to understanding why gradient polymers perform promisingly as icephobic materials. Figure 6a shows the condensation frosting rate in different coatings. After 10 s, condensation frosting covered 95% of the area in silicon and 45% of the area in pV_4_D_4_. Differently, in pPFDA, Grad100, Grad200, and Grad300, only 1.5, 3.6, 3.5, and 1% were covered, respectively. For Grad100, Grad200, and Grad300, 50% of the area was covered after 115, 120, and 280 s, respectively. The frost propagation was evidently delayed over the gradient polymer surfaces.

The differences in condensate shape and distribution were fundamental for the delay. In silicon, it was rather irregular, with the presence of “mother droplets” of 195.44 μm average diameter, completely surrounded with tiny droplets (average diameter of 6.42 μm), creating a quasi-continuous layer of water. In the gradient surfaces, the droplets were relatively uniformly distributed and exhibited a well-defined spherical shape with an average diameter of 62.82 μm and a narrow distribution (Figure S6). Droplets were surrounded by dry zones, where the coating was directly exposed to the air (Figure 6b). Consequently, the condensation frosting propagation followed different mechanisms on both surfaces. On silicon, the propagation happened directionally, continuously, and quickly. The nucleation started at the edges and rapidly propagated in a chain reaction over the entire observable area through the formation of bridges between neighboring drops, a mechanism known as “interdrop ice propagation” studied by Boreyko and Collier.^61^ This mechanism is a consequence of a simultaneous evaporation and instantaneous deposition process from liquid droplets that surround a frozen one. The propagation rate on silicon was 0.28 mm^2^/s, i.e., the observable area was covered in less than 10 s. Although the frost front propagation was not sharply observed, it can be followed as shown in Figure 6c. The condensation frosting consisted of a densely packed ice layer of frozen drops with undisguisable boundaries and a dense dendritic layer on top.

In gradient polymer surfaces, frost formation began sporadically in different droplets across the observed area, proceeding slowly (Figure 6c). The droplets froze individually, very slowly, and no immediate freezing of nearby droplets was triggered; however, an instant out-of-plane growth of dendrites from the tip of the droplet was observed. The dendrite growth occurred faster than the lateral propagation, as a result of a direct desublimation of water vapor from the surroundings to the dendrites.^62,63^ The dendrites seemed to function as water vapor harvesting points: not only the vapor pressure of ice is lower compared to liquid water but also water vapor content may be higher at the top, promoting fast growth while lagging the frost formation at the droplet level.^64^ Neither interdrop ice propagation nor the growth of ice crystals from a frozen drop toward the neighbors, as described by Jung et al.,^65^ was observed to be the propagation mechanism. Heterogeneous nucleation prevalently governed the process, no chain reaction was identified and therefore lacked propagation front.

Droplets’ size and position were constant, and when droplets coalesced with neighbors, larger dry area zones were produced. The low-energy surface and strong pinning maintained a reduced contact area with the substrate while restricting the droplet mobility. This was crucial during the nucleation and propagation process because, with larger and stable dry zones, the propagation thermodynamic barrier increased, consequently, delaying the condensation frosting process. The frost formation occurred at a rate of 0.015, 0.011, and 0.006 mm^2^/s for Grad100, Grad200, and Grad300, respectively. Compared with silicon, that corresponds to a process 18, 25, and 47 times longer, accordingly. The condensation frosting consisted of a non-continuous ice layer conformed by scattered frozen droplets with fragile dendrites on top, and surprisingly, some remaining dry zones, forming an overall airy frost structure (Figure 6c). The mechanism observed in the gradient polymers was noticed to be much slower compared with other materials found in the literature, e.g., inspired by the antiprotein approach.^66^

The systematic trend observed in the gradient polymers was consistent throughout the icephobic assessment: as the top section thickness increases, there is an improvement in icephobicity. Although there are other parameters between the water-coating interaction, such as chemistry, topography, wettability, and thermal conductivity, we hypothesize that the surface energy discontinuity, resulting from variations in crystallographic orientations induced by the top section thickness, is the primary factor responsible for the icephobic properties. Surfaces with contrasting energy surfaces have been reported to affect the water molecule organization and therefore induce a delay in ice nucleation and frost propagation.^67,68^ This was experimentally observed by the differences among the gradient polymers and their comparison with pure pPFDA. Furthermore, the surface energy discontinuity leads to different electrostatic interactions happening between the coating and water molecules before, during, and after freezing. Since one of the main contributions to the ice adhesion mechanism is the electrostatic interaction,^23,52^ a surface that alters these interactions is expected to reduce the ice adhesion. At a molecular scale, the random orientation of the fluorinated groups disrupts the water molecule organization creating a weaker bond toward the surface, and this is macroscopically reflected in lower ice adhesion. This was experimentally observed and also supported by the crystallographic analysis.

## Conclusions

Gradient polymer coatings deposited via iCVD are promising icephobic materials, proved to fully act efficiently in different aspects. These coatings can significantly decrease the ice adhesion independently of the interfacial area since the ice detachment mechanism was found to be toughness-dominated, with a critical length lower than 0.8 cm. Through extensive characterization, we demonstrate that the icephobic properties of this material arise from a surface energy discontinuity due to a random orientation of the fluorinated groups. We demonstrated how tuning the architecture in gradient polymers resulted in a simple and effective approach to induce randomness and thus promote icephobicity by reducing ice adhesion. We presented strong evidence that the icephobic properties originate from an atomistic level and that slight changes at this level have drastic macroscopic consequences.

Furthermore, drop freezing delays longer than 5 h and a lower freezing probability is reported. Microscopy observations revealed that the frost propagation occurs extremely slowly at a rate of 0.006 mm^2^/s due to the limited mobility of the droplet consequence of strong pinning and the prominent presence of dry zones. The developed material demonstrated no correlation between wettability and icephobicity, as the latter was enhanced by the systematic increment of roughness, high contact angle hysteresis, and roll-off angles.

An undiscovered route with high promises is offered by iCVD. Fluorinated compounds which are of high interest but are limited by their solubility, compatibility, and poor stability due to their low control can be managed using this technique. The gradient polymer approach grants outstanding stability and durability against scratches and delamination. This opens new opportunities for monomers showing low adhesion or miscibility problems. Using iCVD, the manufacturing of these coatings is done in one step, in which no pre- or post-treatments are required, presenting a very attractive alternative for industrial applications. An in-depth understanding of the mechanism behind the gradient polymer icephobicity at a molecular level is still undetermined. Molecular dynamic simulation studies, combined with fracture mechanic analysis, will be required to comprehend the atomistic interactions between water-coating molecules and to provide a more detailed insight into the factors influencing the icephobic properties and ice adhesion mechanism. Coupling these coatings with current deicing technologies is a viable alternative that can improve their performance for ice mitigation and icing control.

## Supporting Information Available

Schematic representation of experimental setups; notes and results of XPS analysis; AFM images; notes and results of cross-hatch adhesion test; drop freezing delay time results; in situ XRD during icing plots; notes and images of condensation observations; and droplet size distribution plot (PDF)
