# Supplementary material for: Icephobic Gradient Polymer Coatings Deposited via iCVD: A Novel Approach for Icing Control and Mitigation
Source: ACS Appl Mater Interfaces. 2024 Feb 24;16(9):11901–13. doi: 10.1021/acsami.3c18630 (PMC10921382; doi:10.1021/acsami.3c18630)
Supplement: Supplementary file 1 — am3c18630_si_001.pdf [file am3c18630_si_001.pdf]

Supplementary Information

Icephobic gradient polymer coatings deposited via iCVD: A novel approach for icing control and mitigation

Gabriel Hernández Rodríguez [a], Mario Fratschko [a], Luca Stendardo [b], Carlo Antonini [b], Roland Resel [a], Anna Maria Coclite [a]*

[a] Institute of Solid State Physics, NAWI Graz, Graz University of Technology, 8010 Graz, Austria

[b] Department of Materials Science, University of Milano – Bicocca, via R. Cozzi 55, 20125 Milano, Italy

*Corresponding author: anna.coclite@tugraz.at

Section 0. Icing experimental Setups

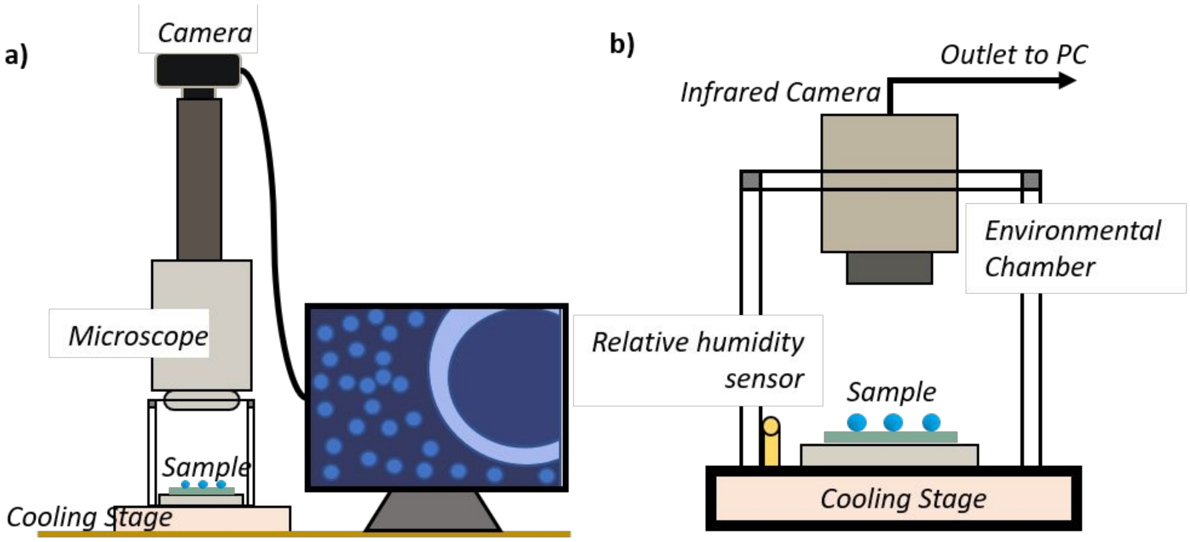

Figure S1.- a) Frost nucleation experimental setup. b) Drop freezing delay experimental setup

Section 1. FT-IR Analysis.

The sharp peaks at 1210 and 1240 cm^{-1} correspond to the asymmetric and symmetric stretching of the fluorinated moieties (–CF_2) and the peak at 1150 cm^{-1} corresponds to the –CF_2–CF_3 end group. The peak at 1740 cm^{-1} corresponds to the carbonyl group (C=O). The peak at 1064 cm^{-1} is associated with the asymmetric Si–O–Si stretching in the siloxane ring. The peak at 1260 cm^{-1} corresponds to the symmetric bending of the Si–CH_3. The weakening of the peaks associated with vinyl groups (–C=C–) at 1290 cm^{-1} and 1590 cm^{-1} is an indication of the consumption of available vinyl groups during the polymerization and copolymerization.

Section 2. X-ray photoelectron spectroscopy (XPS)

	O(%)	*Si(%)*	*F(%)*	*C(%)*	*C/F*
pPFDA	*5.25*	*0.56*	*49.53*	*44.66*	*0.90*
Grad100	*5.19*	*0.89*	*48.21*	*45.71*	*0.94*
Grad200	*5.42*	*0.01*	*48.14*	*46.43*	*0.96*
Grad300	*5.27*	*0.48*	*50.6*	*43.56*	*0.86*

No significant differences can be seen from the elemental composition and the peak shape of the C1s and F1s peaks. No Si could be detected. This indicates that in the top 10 nm of the polymers, there is no pV_4D_4 (as expected). The C/F is not significantly different among the 4 polymers.

Section 3. Atomic force microscopy (AFM)

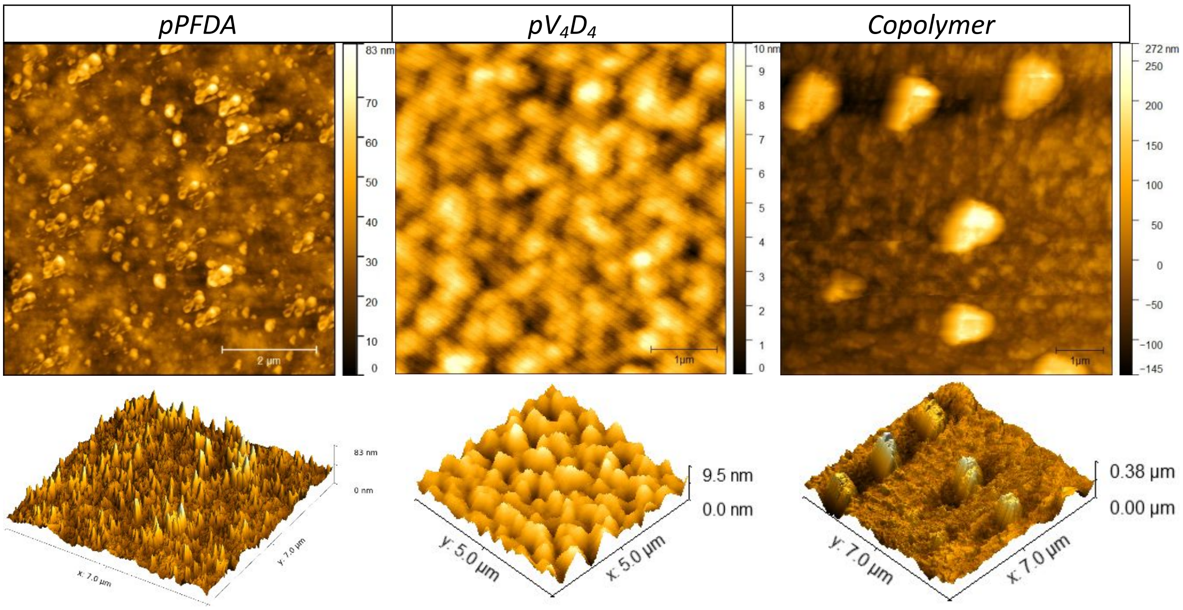

Figure S2.- Atomic force imaging of pPFDA, pV_4D_4 and the copolymer $p(V_4D_4$-co-PFDA).

Section 4. Cross-hatch adhesion

According to the ASTM D3359 classification of adhesion for method B, the gradient coatings are rated with 5B, corresponding to the strongest adhesion observable, whose description states: "The edges of the cuts are completely smooth; none of the squares of the lattice is detached.". In contrast with the gradient polymer coatings, pPFDA and pV_4D_4 rated with 2B, whose description states: "The coating has flaked along the edges of the cuts partly or wholly in large ribbons, and/or it has flaked partly or wholly on different parts of the squares. A crosscut area greater than 15% but not greater than 35%, is affected.". As expected, after the test was repeated five times, most of the coating was detached.

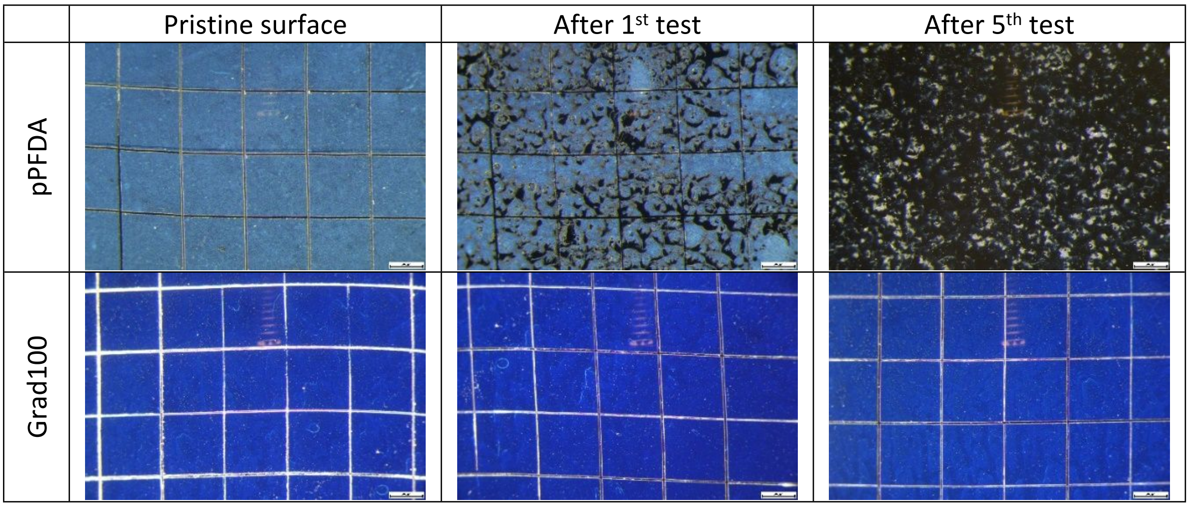

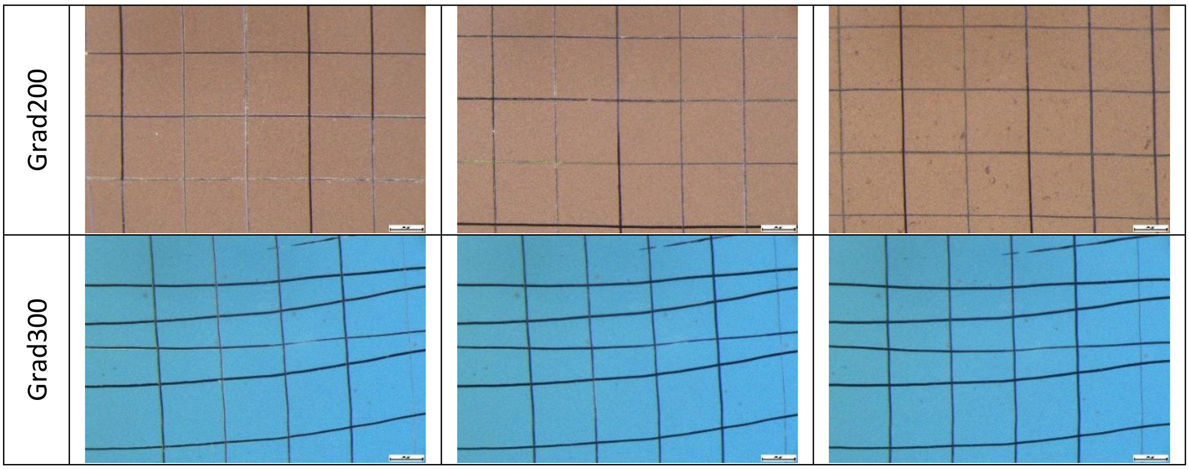

Figure S3.- Optical microscopy images of the sample before and after the cross-hatch adhesion test. No damage was visible in the gradient surfaces after the tests.

Section 5. Drop Freezing Delay

	Grad100			*Grad200*			*Grad300*		
	<10%	*50%*	*>70%*	*<10%*	*50%*	*>70%*	*<10%*	*50%*	*>70%*
-20°C	*7240 s*	*195.87 s*	*155.87 s*	*10800 s*	*229.62 s*	*180.67 s*	*18000 s*	*493.27 s*	*208.35 s*
-25°C	*8.48 s*	*3.73 s*	*0.53 s*	*33.8 s*	*4.40 s*	*1.20 s*	*62.94 s*	*6.76 s*	*2.7 s*
-30°C	*2.36 s*	*2.92 s*	*0.00 s*	*2.50 s*	*0.03 s*	*0.00 s*	*3.17 s*	*0.86 s*	*0.74 s*

Section 6. In-situ XRD during icing

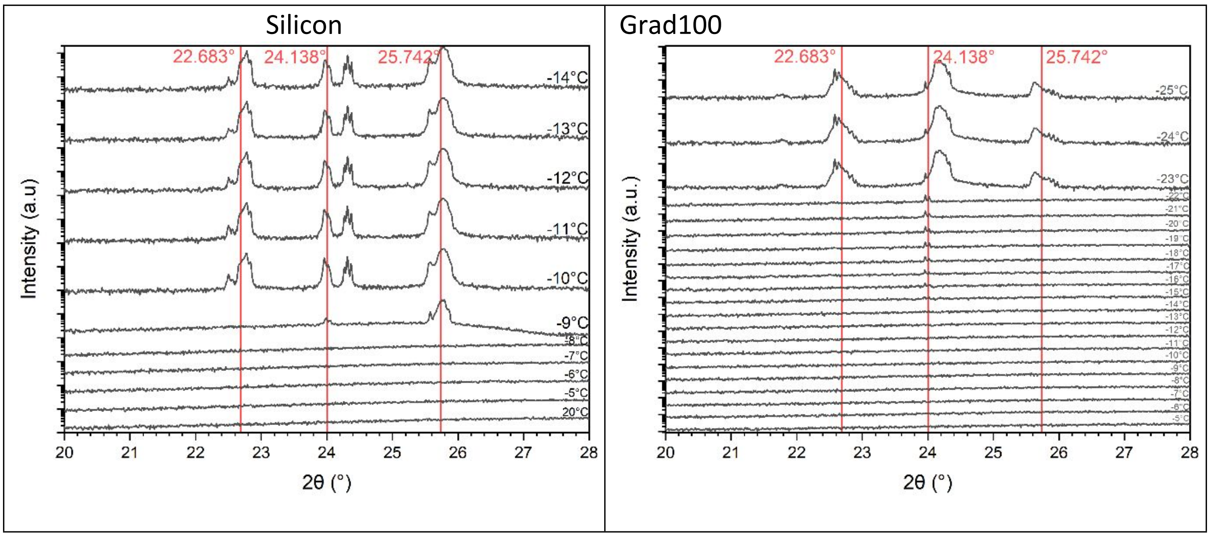

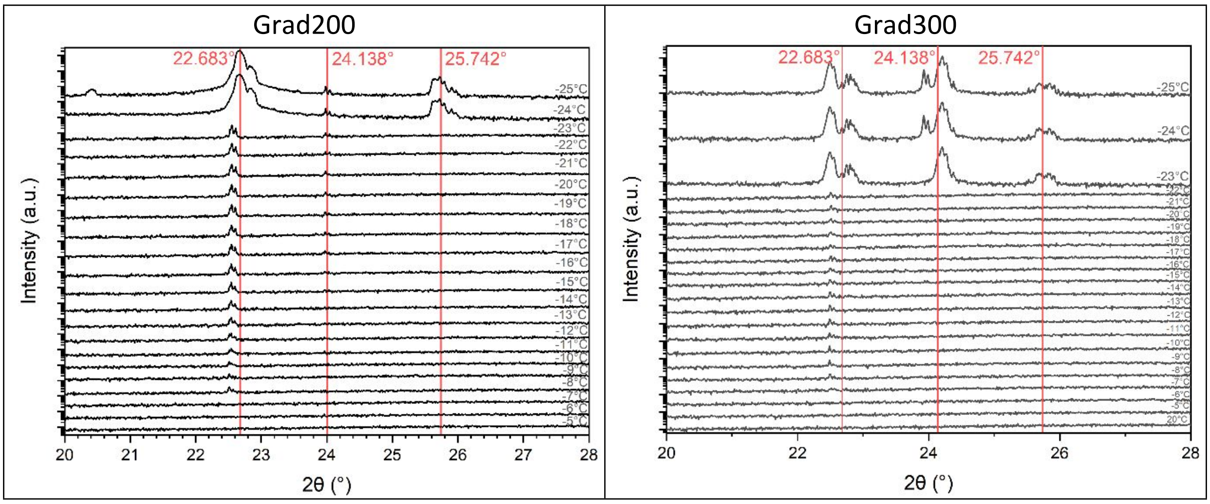

Figure S4.- Difractograms of in-situ XRD during icing.

Section 7. Condensation

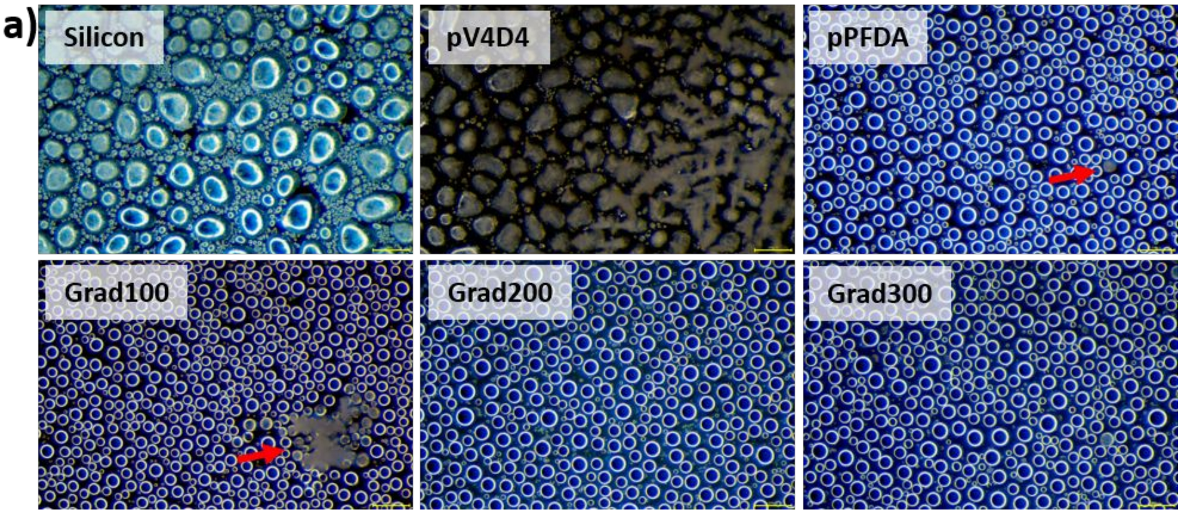

Figure S5.- Water condensation on different surfaces at a temperature of -20°C

Microscope images of the different surfaces at -20°C. The silicon and pV_4D_4 sample surface show an advanced state of condensed water freezing. This can be identified by the milky appearance of the drops. The red arrows in the pPFDA and grad100 images indicate the freezing nucleation points. In contrast, grad200 and grad 300 surfaces are clear from freezing nucleation points.

Section 8. Frost rate and propagation mechanism

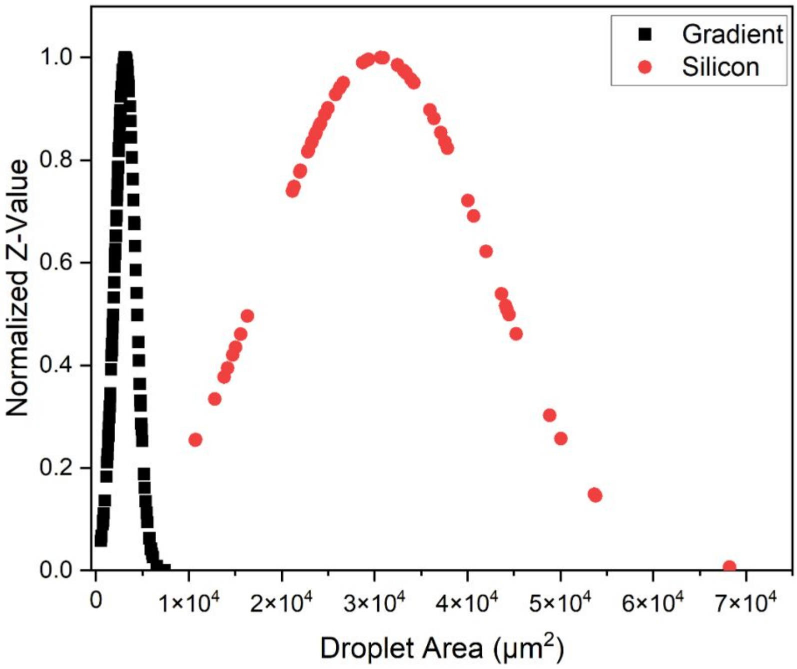

Figure S6.- Comparison of the droplet size distribution observed in the gradient polymer vs silicon substrate.
